# The effectiveness of virtual reality based interventions for symptoms of anxiety and depression: A meta-analysis

**DOI:** 10.1038/s41598-018-28113-6

**Published:** 2018-07-09

**Authors:** Liviu A. Fodor, Carmen D. Coteț, Pim Cuijpers, Ștefan Szamoskozi, Daniel David, Ioana A. Cristea

**Affiliations:** 1 International Institute for The Advanced Studies of Psychotherapy and Applied Mental Health, Babeș-Bolyai University, Cluj-Napoca, Romania; 2 Evidence Based Psychological Assessment and Interventions Doctoral School, Babeș-Bolyai University, Cluj-Napoca, Romania; 3 Department of Clinical Psychology and Psychotherapy, Babeş-Bolyai University, Republicii Street 37, 400015 Cluj-Napoca, Romania; 4 Department of Clinical, Neuro and Developmental Psychology, Vrije Universiteit, Amsterdam, The Netherlands; 5 Amsterdam Public Health Research Institute, Vrije Universiteit, Amsterdam, The Netherlands; 6 Department of Applied Psychology, December 21 1989 Street 128, Babeș-Bolyai University, Cluj-Napoca, Romania; 7 Department of Oncological Sciences, Icahn School of Medicine at Mount Sinai, New York, USA; 8 Meta-Research Innovation Center at Stanford, Stanford University, Stanford, California USA

## Abstract

We report a meta-analysis of virtual reality (VR) interventions for anxiety and depression outcomes, as well as treatment attrition. We included randomized controlled trials comparing VR interventions, alone or in combination, to control conditions or other active psychological interventions. Effects sizes (Hedges’ *g*) for anxiety and depression outcomes, as post-test and follow-up, were pooled with a random-effects model. Drop-outs were compared using odds ratio (OR) with a Mantel-Haenszel model. We included 39 trials (52 comparisons). Trial risk of bias was unclear for most domains, and high for incomplete outcome data. VR-based therapies were more effective than control at post-test for anxiety, g = 0.79, 95% CI 0.57 to 1.02, and depression, g = 0.73, 95% CI 0.25 to 1.21, but not for treatment attrition, OR = 1.34, 95% CI 0.95 to 1.89. Heterogeneity was high and there was consistent evidence of small study effects. There were no significant differences between VR-based and other active interventions. VR interventions outperformed control conditions for anxiety and depression but did not improve treatment drop-out. High heterogeneity, potential publication bias, predominant use of waitlist controls, and high or uncertain risk of bias of most trials question the reliability of these effects.

## Introduction

Virtual reality (VR) has garnered significant attention as a cost-effective tool for delivering psychological treatments^1^. Virtual reality exposure (VRE) in particular is considered an effective treatment for several anxiety disorders^2^, on par with *in vivo* exposure/IVE^3,4^, though doubts were expressed about the quality of this evidence^5^.

While many narrative reviews and commentaries focused on VR interventions, only three systematic reviews with meta-analyses examined their efficacy in randomized controlled trials/RCTs^4,6,7^ and they present certain shortcomings. Included trials were published through 2014 the latest, and many more trials have been conducted since, given VR technology has become more accessible. Outcomes other than anxiety were scarcely analyzed, though data on some of these has been accruing. The effects of VR interventions on treatment attrition remained unclear, with some speculation of possible superiority^1,5,8^, but no assessment in a meta-analysis.

Only one meta-analysis^7^ considered heterogeneity between effect sizes (ESs), but did so only descriptively, without providing a quantification. Assessment of quality^6,7^ relied on mixed and potentially inadequate tools that included items not linked to any type of trial bias (e.g., treatment fidelity)^9^, thereby potentially confounding the relationship between study quality and treatment effects. Only one meta-analysis^7^ considered publication bias, with conflicting results between the assessment methods used (Egger’s test and fail-safe N). Moreover, many VR trials are conducted on a small number of participants, which exposes meta-analyses to “small study effects”^10^, the notion that smaller studies show different, often larger, treatment effects than large ones. Few potential moderators were examined, with generally contradictory results regarding treatment intensity, or the type of comparison group. One yet uninvestigated potential moderator regards the involvement of developers of VR tools and interventions in the trials, as these are often for-profit developments.

Consequently, we report a meta-analysis for the effectiveness of VR-enhanced interventions in RCTs, for symptoms of anxiety and depression, as well as treatment attrition, along with assessment of risk of bias, heterogeneity, and potential moderators.

## Methods

### Identification and selection of studies

A literature search of PubMed, PsycInfo, EMBASE and Cochrane Central Register of Controlled Trials databases was conducted through May, 2015, updated in March, 2016 and subsequently August 2017, using the keywords “virtual reality”, “therapy”, “exposure”, “intervention”, “treatment” and a filter for randomized trials (Supplementary Method). We also searched the references from the most recent systematic reviews and meta-analyses.

Studies were included if they were a) RCTs comparing b) a VR-enhanced intervention to a control or an active psychological intervention for c) adults, d) measuring outcomes related to depression and anxiety, and e) published in peer-reviewed journals. We included studies comparing a VR-enhanced condition with controls (e.g., waitlist, placebo, treatment-as-usual) or active conditions not employing VR. Similarly to Turner & Casey (2014), the latter were defined as established interventions involving active, psychologically therapeutic mechanisms of action (e.g., CBT, IVE). No language restrictions were employed. One researcher screened all abstracts and full-texts of RCTs were recovered. Two independent researchers independently examined full-texts and selected eligible RCTs. Disagreements were resolved by discussion and consultation with a third author until consensus was reached.

### Risk of bias and data extraction

We used four criteria from the *Risk of Bias* (RoB) assessment tool, developed by the Cochrane Collaboration^11^, which assesses possible sources of bias in RCTs. The following domains were rated: a) the adequate generation of allocation sequence, b) the concealment of allocation to conditions, c) the prevention of knowledge of the allocated intervention (blinding of assessors) and d) the adequate addressment of incomplete outcome data. Blinding of assessors was rated as low risk if the trial described proper methods of ensuring it or if all relevant outcome measures were self-report, thus not requiring the direct interaction with an assessor. This choice was made as we expected most outcomes to be reported on self-report scales, and there is currently no standard as to how to rate these in terms of blinding. Domain d) was assessed as low risk if there were all randomized participants were included in the analysis, either through the use on an intent-to-treat (ITT) approach or when complete data was available. We also computed an overall RoB score for each study by awarding 1 point for each bias source rated as *low* risk.

We extracted a series of variables from the included studies, detailed in Table 1 for further use in moderator analyses. Details about the interaction with the virtual environment were extracted from the methods sections describing the intervention or the technology used. For each trial, we noted which elements the interaction with the VR environment relied upon (e.g., visual, sound, haptic) and (2) whether or not the authors had explicitly assessed sense of presence or immersion in the trial with validated or *ad hoc* instruments. We also quantified the first component by tabulating the number of interaction elements each study employed, as a very crude indicator of the degree of interaction.

**Table 1:** List of variables that were extracted from the included studies

Variable name	Coding categories
**Categorical variables**	
Study location	North America (N. America)
	Europe (EU)
VR program developer as an author of the study	Yes
	No
Recruitment pool	Community volunteers
	Clinical setting
	Army enlisted personnel
Type of control or comparison group	Other (Placebo/Relaxation/Treatment-as-usual)
	Waitlist
	CBT (cognitive-behavioral therapy)
	IE (imaginal exposure)
	IVE (*in-vivo* exposure)
Type of VR-enhanced intervention	VRCBT (VR-enhanced CBT)
	VRE (exposure in virtual reality)
Type of anxiety disorder (only for anxiety symptoms)	Flight anxiety
	Panic disorder
	PTSD
	Social anxiety
	Specific phobia
Risk of bias for incomplete outcome data	High/Unclear risk of bias
	Low risk of bias
**Continuous variables**	
Publication year	
Number of subjects randomized to the VR-enhanced group	
Number of drop-outs from the VR-enhanced group	
Participant’s mean age	
Number of VR sessions	
Session duration	as measured in minutes
Overall VR therapy duration (weeks)	as measured in weeks
Risk of bias score	coded as the total number of criteria at low risk of bias for each included study
Number of elements involved in the interaction with the virtual environment	

.

The involvement of a developer was coded using the information available in each trial, at the section of the method that described the VR therapy package used. If authors of the VR package were not listed in the original article, we independently searched the web for the specific VR program or package used in order to identify its authors. Risk of bias assessment and data extraction were performed by two independent researchers and disagreements were discussed and resolved until consensus was reached.

### Meta-analyses

We computed and pooled the individual ESs with Comprehensive Meta-Analysis (CMA version 3.3.070) and Stata (Stata SE, version 15).

For anxiety and depression, we calculated the standardized mean difference (SMD) at post-test and follow-up, by subtracting the mean score of the comparison group (control or active treatment) from the mean score of the VR-enhanced group, and dividing the result by the pooled standard deviation of the two groups. Positive SMDs thus reflect superiority of the VR-enhanced condition. We report the indicator corrected for small sample bias^12^, Hedges’ *g*. We also transformed the SMD into number needed to treat (NNT), using the formula of Kraemer & Kupfer^13^. The NNT represents the number of patients that would have to be treated to generate one additional positive outcome^14^.

Given the considerable variability among outcomes measures, we grouped them into anxiety and depressive symptoms. These included all such outcomes, whether measured by general or disorder-specific scales or subscales. As anxiety outcomes were sometimes measured for individuals without an anxiety disorder, we also conducted sensitivity analyses restricted to patients with one such disorder, diagnosed with a clinical interview or by use of a cut-off at a symptom scale. When a study used multiple measures from the same category, the average ES was computed using the CMA procedure^15^ that assumes a correlation of 1 between outcomes. Since the correlation is probably less than 1, this approach is conservative^16^. ITT data were preferred where available. If means and standard were not available, we calculated the SMD from other statistics available in the study, such as *t*-values or exact *p-*values, using the standard formulae in the program^15^. If data was still insufficient for ES calculation, a request was sent to the study authors.

Drop-outs were defined as all randomized participants not finishing treatment, regardless of the reasons. Odds ratio (ORs) indicated the odds of participants dropping out from the VR versus the comparison group, with sub-unitary ORs indicating smaller odds for drop-out in the VR group.

We conducted separate meta-analyses for VR-enhanced therapy versus control, and respectively versus other active psychological treatments. Continuous outcomes (anxiety, depression) were pooled with a random effects model using the inverse-variance DerSimonian and Laird method^17^. For dichotomous outcomes, given we expected small trials, with some reporting few or no drop-outs, we used both the fixed effect Mantel-Haenszel method^18,19^ with a continuity correction of 0.5 for zero counts, as well as Peto’s method^20^, as previously recommended^21,22^. Trials with zero drop-outs in both arms were excluded, due to concerns they might significantly inflate bias particularly in small trials^21^. We conducted sensitivity analyses excluding outliers and, respectively, excluding studies with a small number (N) of participants. Outliers were defined as studies in which the pooled ES’s 95% CI was outside the 95% CI of the pooled ES (on both sides). We used an arbitrary cut-off of at least 25 randomized participants per arm to for the analysis excluding small N studies. Though power calculations might differ from trial to trial, larger N trials are at least more precise in estimating the intervention effect^23^.

Heterogeneity was assessed with the *I*^2^ statistic, with values of 25%, 50% and respectively 75% indicating low, moderate and high heterogeneity^24^. We calculated 95% confidence intervals (CI) around *I*^2 ^^25^, using the non-central χ^2^-based approach^26^. For categorical moderators, we conducted subgroup analyses using the mixed effects model, which uses a random-effects model within subgroups and a fixed-effects one across subgroups^15^. For continuous moderators, meta-regression analyses employed a restricted maximum likelihood model with the Knapp-Hartung method^15^.

We investigated small study effects and publication bias using a variety of methods. We resorted to visual inspection of the funnel plot, and contour enhanced funnel plots^27^, where contour lines indicate regions where a test of treatment effects was significant for various established levels for statistical significance. We also employed statistical tests for small study effects. In the case of continuous outcomes, we conducted Egger’s test^28^ for the asymmetry of the funnel plot and corresponding Galbraith plots^29^ if the test indicated significant asymmetry. We also used the trim and fill procedure^30^ as a complementary method to adjust for potential publication bias or small study effects. For drop-out rates, as these were binary outcomes pooled with the ORs, we used the Harbord test^31^, which regresses Z/sqrt(V) against sqrt(V), where Z is the efficient score and V is Fisher’s information (the variance of Z under the null hypothesis).

### Data availability

The datasets generated and analysed during the current study are available in the Figshare repository, 10.6084/m9.figshare.5675407.

## Results

### Selection and inclusion of studies

**Figure 1:**
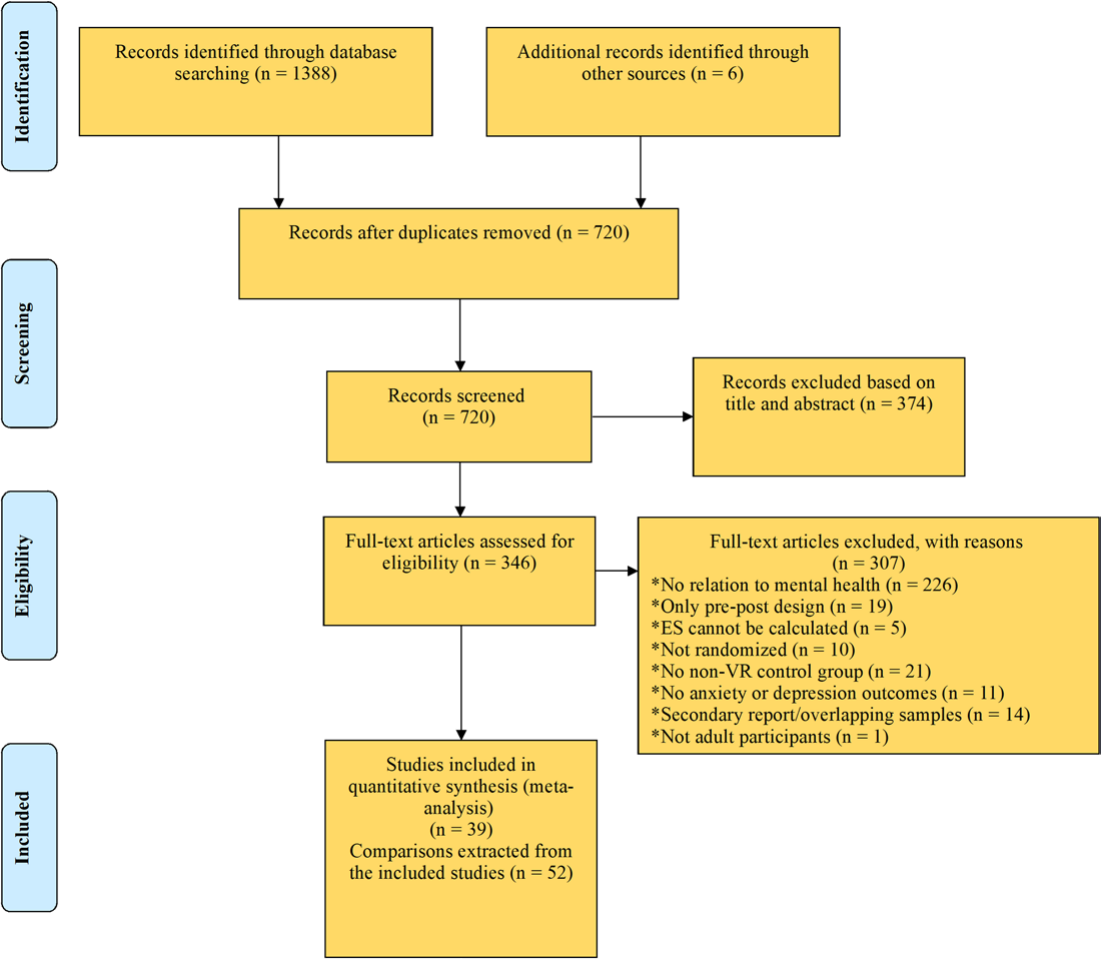
PRISMA flow-diagram of the study selection process.

The search generated 1394 records (720 after duplicate removal). We excluded 374 records based on abstract inspection and examined the full-texts for 346 articles. Figure 1 reports the flowchart of the inclusion process following the PRISMA guidelines^32^. Subsequently, 42 trials met our inclusion criteria, six of which had insufficient data for ES calculation. Following contact with the original authors, we obtained data for one study^33^. For two others^34,35^ the author confirmed the samples overlapped with those from larger included studies. For 3 remaining trials, authors did not provide data, thus leaving a total of 39 trials in the meta-analysis (Supplementary Result).

### Characteristics of included studies

The 39 RCTs included 52 relevant comparisons, with 869 participants in the VR-enhanced condition, and 1122 in the control or active treatments ones. The most frequent conditions were anxiety and anxiety-related (e.g., PTSD) disorders (31 studies). The most frequently used VR therapy was VRE (in 21 out of the 39 RCTs), followed by VRCBT (in 19 out of the 39 RCTs). The number of VR sessions ranged from 1 to 16. The most used VR device was the head-mounted display (HMD) (35 studies). Apart from visual feedback, the majority of studies included sound (27 studies) or some form of navigation (18 studies). Only 6 trials explicitly assessed presence or immersion in the virtual environment. In most cases, developers of the VR program used were also among the authors (27 studies) (Table 2; Supplementary Table S1).

**Table 2:** Selected characteristics of included studies of VR-enhanced interventions.

Study	Condition^a^	Sample^b^	Recr.^c^	N_rand_ VR^d^	N_sess_ VR^e^	VR_weeks_^f^	VR psy interv.^g^	Ctrl^h^	VR system^i^	VR dev^j^	Prov^k^
Anderson, 2013	Social anxiety	DSM-IV-TR	Comm	30	8	8	VRCBT	WL; CBT	HMD	N	US
Banos, 2011	Mixed disorders	DSM-IV-TR	Comm	25	5	9	VRCBT	CBT	VR room	Y	ES
Botella, 2007	PD + AG	DSM-IV, ADIS-IV	Comb	12	6	9	VRCBT	WL; CBT	HMD	Y	ES
Botella, 2016	Spider Phobia	DSM-IV-TR	Comm	32	1	0.14	VRE	IVE	HMD	Y	ES
Bouchard, 2016	Social Anxiety	DSM-V	Comm	17	8	14	VRCBT	WL; CBT	HMD	Y	CA
Choi, 2005	PD + AG	DSM-IV	Clin	20	3	4	VRCBT	CBT	HMD	Y	KR
Emmelkamp, 2002	Acrophobia	DSM-IV/BAT	Comm	17	3	3	VRE	IVE	HMD	N	NL
Gaggioli, 2014	Stress	VAS-A	Comm	40	8	5	VRCBT	WL; CBT	HMD	Y	IT
Garcia-Palacios, 2002	Spider Phobia	FSQ > 97, DSM-IV	Comm	12	4	2.5	VRE	WL	HMD	Y	ES
Kampmann, 2016	Social Anxiety	DSM-IV-TR	Comm	20	7	5	VRE	WL; IVE	HMD	Y	NL
Krijn, 2004	Acrophobia	DSM-IV, BAT	NR	17	3	3	VRE	WL	HMD/CV	N	NL
Lau, 2010	Ward orient	Psych diagn.	Clin	27	1	0.14	VRE	TAU	PC	N/R	CN
Malinvaud, 2016	Tinnitus	Subj. tinnitus	Clin	61	8	8	VRE	CBT	HMD	Y	FR
Maltby, 2002	Flight Anxiety	DSM-IV	Comm	25	5	3	VRE	PLB	HMD	Y	US
McLay, 2011	PTSD	MINI/CAPS > 40	Army	10	8,8	10	VRE	TAU	HMD	Y	US
McLay, 2017	PTSD	DSM-IV	Army	43	10,28	9	VRE	IE	HMD	Y	US
Meyerbroeker, 2013	PD + AG	DSM-IV-TR	NR	27	6	10	VRCBT	CBT	HMD/CV	N	NL
Michaliszyn, 2010	Spider Phobia	DSM-IV, BAT	Comm	16	6	8	VRE	IVE	HMD	N	CA
Miyahira, 2012	PTSD	CAPS, PDS	Army	29	9	5	VRCBT	WL	HMD	N	US
Muhlberger, 2001	Flight Anxiety	DSM-IV	Comm	15	1	0.14	VRE	RLX	HMD	N	DE
Muhlberger, 2003	Flight Anxiety	DSM-IV	Comm	26	1	0.14	VRCBT	CT	HMD	N	DE
Pelissolo, 2012	PD + AG	DSM-IV	Clin	43	12	12	VRE	CBT	HMD	N	FR
Pitti, 2008	PD + AG.	CIDI	Clin	18	11	11	VRCBT	CBT	CV	N	ES
Ready, 2010	PTSD	CAPS > 60	Clin	6	10	N/R	VRE	PLB	HMD	Y	US
Reger, 2016	PTSD	DSM-IV-TR	Army	54	8	10	VRE	WL; IE	HMD	Y	US
Riva, 2003	BED	DSM-IV	Clin	9	10	6	VRCBT	WL; CBT	HMD	Y	IT
Riva, 2006	Severe Obesity	BMI > 41	Clin	57	9	6	VRCBT	WL; CBT	HMD	Y	IT
Robillard, 2010	Social Anxiety	DSM-IV-TR	NR	14	16	NR	VRCBT	WL; CBT	HMD	Y	CA
Rothbaum, 1995	Acrophobia	AQ (screening)	Comm	12	7	8	VRE	WL	HMD	Y	US
Rothbaum, 2000	Flight Anxiety	DSM-IV	Comm	15	4	6	VRCBT	WL; CBT	HMD	Y	US
Rothbaum, 2006	Flight Anxiety	DSM-IV	Comm	41	4	6	VRCBT	CBT	HMD	Y	US
Rus-Calafell, 2013	Flight Anxiety	DSM-IV	Comm	7	6	3	VRE	IE	HMD	Y	ES
Stetz, 2011	Stress	PCL-M	Army	30	3	0.42	VRE	RLX	Screen	Y	US
Thompson, 2011	Tiredness/Mood	No diagnostic	Comm	12	10	2.5	VRE	RLX; IE	HMD	Y	UK
Tortella-Feliu, 2011	Flight Anxiety	DSM-IV	Comm	19	6	3	VRE	IE	HMD	Y	ES
Triscari, 2015	Flight Anxiety	MCMI-III, DSM-V	Comm	21	3	10	VRCBT	CBT	N/R	Y	IT
Vincelli, 2003	PD + AG.	DSM-IV	Clin	4	8	N/R	VRCBT	WL; CBT	HMD	Y	IT
Wallach, 2009	Social Anxiety	PSA symptoms	Comm	34	8	12	VRCBT	WL; CBT	HMD	N	IL
Wiederhold, 2001	Flight Anxiety	DSM-IV	Comm	20	6	8	VRE	IE	HMD	N/R	US

^a^PD = panic disorder; ED = eating disorder; orient = orientation; BED = binge eating disorder; AG = agoraphobia; PTSD = post-traumatic stress disorder. Mixed disorders include PTSD, pathological grief and adjustment disorders;

^b^Sample selection; DSM = Diagnostic and Statistical Manual of Mental Disorders; ADIS = Anxiety Disorders Interview Schedule; BAT = behavioral approach test; VAS-A = Visual Analogue Scale for Anxiety; FSQ = Fear of Spiders Questionnaire; Psych diagn. = 1^st^ time admission in a psychiatric ward; Subj. tinnitus = subjective tinnitus; MINI = Mini-International Neuropsychiatric Interview; CAPS = Clinician Administered PTSD Scale; PDS = PTSD Diagnostic Scale; CIDI = Composite International Diagnostic Interview; BMI = Body Mass Index; AQ = Acrophobia Questionnaire; PCL = PTSD CheckList – Military Version; MCMI = Millon Clinical Multiaxial Inventory; PSA Symptoms = public speaking anxiety symptoms (psychiatric evaluation);

^c^Comm = recruited from community samples; Clin = recruited from clinical samples; NR = not reported;

^d^N_rand_ VR = number of participants randomized to the VR-enhanced treatment;

^e^N_sess_ VR = number sessions of VR-enhanced treatment;

^f^VR_weeks_ = the duration in weeks of the VR-enhanced treatment;

^g^VR psy tx = type of VR-enhanced psychological treatment; VRE = VR-enhanced exposure; VRCBT = VR-enhanced cognitive behavioral therapy;

^h^Ctrl= control/comparison intervention; IVE = *in vivo* exposure; IE = imaginal exposure; RLX = relaxation; CBT = cognitive behavioral therapy; PLB = placebo; CT = cognitive therapy; WL = waitlist; TAU = treatment-as-usual;

^i^HMD = head-mounted display; CV = Cave-type system;

^j^VR dev = VR developers are among the study authors; Y = yes; N = no;

^k^Prov, provenience; CN = China; NL = Netherlands; ES = Spain; US = United States; KR = South Korea; IT = Italy; FR = France; CA = Canada; DE = Germany; IL = Israel.

### Risk of bias of the included studies

**Figure 2:**
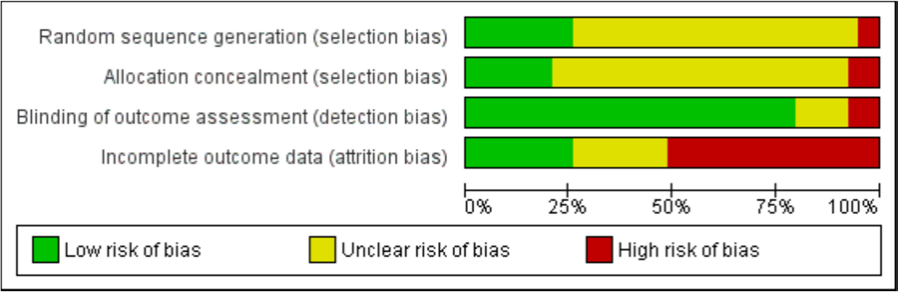
Risk of bias graph: review authors’ judgments about each risk of bias item presented as percentages across all included studies.

Most trials had uncertain or high risk of bias for three domains. Four RCTs had low RoB on all four domains. Nineteen studies were rated low RoB in only one domain. For sequence generation and allocation concealment, the majority of trials (27 and respectively 28) did not provide any information to enable assessment. For blinding, only seven studies employed actual blinding of outcome assessors and 25 studies used exclusively self-report measures. For incomplete outcome data, 20 studies did not employ ITT analyses, and 9 studies did not include enough information to assess this domain. For this domain, we conducted additional subgroup analysis contrasting trials with low RoB versus the rest. Trials with high and unclear RoB were combined since given the ubiquity of treatment drop-out in RCTs, the lack of any mention of ITT strategies makes it very likely that none had been employed. For 3 trials, the number of drop-outs in one arm was unclear (Table S2) (Fig. 2, Supplementary Figure S1).

### Main results

#### VR-enhanced therapy compared to a control condition

**Figure 3:**
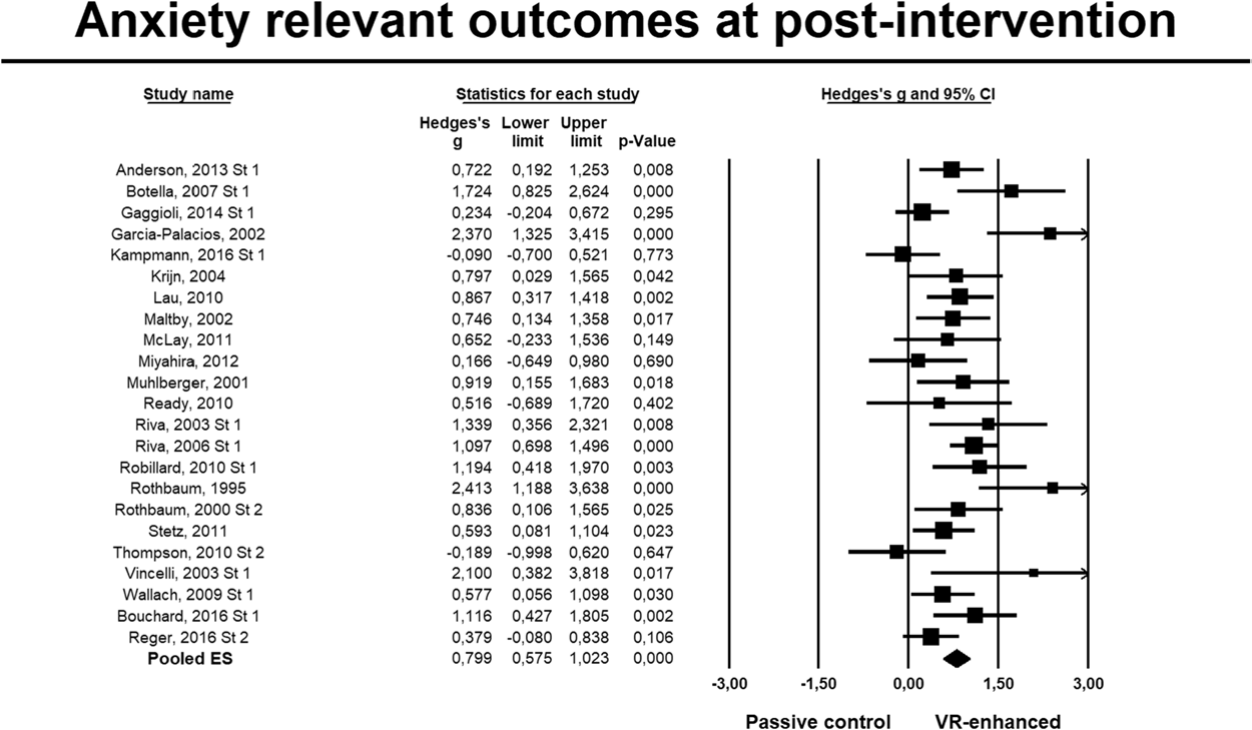
Forest plot: Standardized mean differences post-test for VR-enhanced therapy versus control conditions for anxiety outcomes.

For *anxiety* outcomes (Fig. 3), twenty-three RCTs were pooled, *g* = 0.79, 95% CI 0.57 to 1.02, NNT = 2.36, with substantial heterogeneity (*I*^2^ = 59%, 95% CI 35 to 74). Analyses restricted to participants with an anxiety disorder (17 comparisons) led to slightly smaller estimates: *g* = 0.72, 95% CI 0.51 to 0.94, NNT = 2.56, with similarly substantial heterogeneity (*I*^2^ = 58%, 95% CI 28 to 76). Exclusion of three potential outliers led to a small decrease, *g* = 0.73, 95% CI 0.55 to 0.92, and reduced heterogeneity (*I*^2^ = 36%; 95% CI 0 to 63). Only 7 trials had at least 25 participants randomized in each arm. Their aggregate ES was *g* = 0.64, 95% CI 0.39 to 0.88, and heterogeneity was still present (*I*^2^ = 42%; 95% CI 0 to 76) (Table 3).

**Table 3:** VR-enhanced therapy vs. passive control contrast, post-treatment.

Anxiety symptoms		N	g^a^	95% CI	*I* ^2^	I^2^ 95% CI	NNT	p^b^
All studies		23	0.79	0.57 to 1.02	59	35 to 74	2.36	
Outliers excluded^c^		20	0.73	0.55 to 0.92	36	0 to 63	2.54	
Only studies with >25 randomized per arm		7	0.64	0.39 to 0.88	42	0 to 76	2.86	
Only studies involving anxiety disorders		17	0.72	0.51 to 0.94	58	28 to 76	2.56	
**Subgroup analyses** ^**d**^								
Country	N. America	11	0.74	0.49 to 1.00	31	0 to 66	2.50	0.560
	EU	10	0.90	0.43 to 1.38	77	57 to 87	2.10	
VR program author	N	5	0.64	0.36 to 0.93	0	0 to 79	2.86	0.292
among author pool^e^	Y	17	0.87	0.56 to 1.18	68	48 to 81	2.16	
Recruitment^f^	Army	4	0.45	0.15 to 0.75	0	0 to 85	4.00	*0.0*2*0*
	Clinic	5	1.04	0.75 to 1.34	0	0 to 79	1.86	
	Community	11	0.76	0.38 to 1.13	71	46 to 84	2.44	
Control group	Other (PLB/RLX/TAU)	7	0.63	0.38 to 0.89	0	0 to 71	2.91	0.188
	WL	16	0.90	0.60 to 1.21	68	47 to 81	2.10	
Experimental	VRCBT	11	0.87	0.58 to 1.16	52	5 to 76	2.16	0.536
intervention	VRE	12	0.73	0.38 to 1.07	64	34 to 81	2.54	
Type of anxiety disorder	Flight anxiety	3	0.82	0.42 to 1.22	0	0 to 90	2.28	*0.007*
	Panic disorder	2	1.80	1.01 to 2.60	0	N/A^i^	1.25	
	PTSD	4	0.39	0.04 to 0.74	0	0 to 85	4.59	
	Social anxiety	5	0.67	0.25 to 1.09	58	0 to 84	2.75	
	Specific phobia	3	1.79	0.64 to 2.94	75	17 to 92	1.25	
Incomplete outcome data RoB^j^	High/Unclear	16	0.83	0.60 to 1.06	39	0 to 67	2.26	0.797
	Low	7	0.76	0.26 to 1.26	76	48 to 88	2.44	
**Depressive symptoms**								
All studies		10	0.73	0.25 to 1.21	71	45 to 85	2.54	
Outliers excluded^g^		9	0.60	0.19 to 1.01	62	21 to 82	3.05	
**Subgroup analyses**								
Country	N. America	5	0.69	0.25 to 1.13	44	0 to 79	2.67	0.672
	EU	5	0.93	−0.08 to 1.94	83	61 to 92	2.04	
Recruitment^h^	Army	2	0.32	−0.28 to 0.92	45	N/A	5.56	
	Clinic	3	2.21	0.66 to 3.77	67	0 to 90	1.13	0.066
	Community	3	0.23	−0.53 to 0.99	73	8 to 92	7.69	
Control group	Other(PLB/RLX/TAU)	2	0.87	−0.28 to 2.03	54	N/A	2.16	0.814
	WL	8	0.72	0.16 to 1.28	76	52 to 88	2.56	
Experimental	VRCBT	6	1.01	0.34 to 1.67	69	27 to 87	1.91	0.197
intervention	VRE	4	0.38	−0.29 to 1.06	71	16 to 90	4.72	
Incomplete outcome data RoB	High/Unclear	6	0.81	0.31 to 1.30	46	0 to 79	2.30	0.874
	Low	4	0.72	−0.23 to 1.68	85	62 to 94	2.56	

^a^All results are reported with Hedges’ *g*, using a random effects model. Positive effect indicates superiority of the VR-enhanced therapy over passive control groups.

^b^The *p* levels in this column indicate whether the difference between the ESs in the subgroups is significant (significant results are marked with italic).

^c^Outliers were defined as studies in which the 95% CI was outside the 95% CI of the pooled studies (Kampmann, 2016 St.1; Garcia-Pallacios, 2002; Rothbaum, 1995).

^d^Subgroup analysis were conducted using a mixed effects model. Only subgroups with at least 2 studies were included.

^e^One study (Lau, 2010) did not contain information about this moderator.

^f^Two studies (Krijn, 2004, Robillard, 2010 St.1) did not contain information about this moderator.

^g^Outliers: Vincelli, 2003 St.1.

^h^One study (Robillard, 2010 St.1) did not contain information about this moderator.

^i^Confidence intervals around *I*^2^ cannot be calculated if there are less than 3 groups.

^j^RoB: Risk of Bias.

For *depression,* ten RCTs were pooled, *g* = 0.73, 95% CI 0.25 to 1.21, NNT = 2.54, with high heterogeneity (*I*^2^ = 71%, 95% CI 45 to 85). Exclusion of one outlier resulted in a sizable decrease, *g* = 0.60, 95% CI 0.19 to 1.01, *I*^2^ = 62%. Only one trial^36^ had at least 25 participants randomized in each arm.

Follow-up outcomes were only reported in two RCTs for anxiety and in one for depression.

Seventeen trials reported non-zero *drop-outs* in at least one group and nine trials reported zero drop-outs in both groups (Supplementary Table S2). Drop-out rates did not significantly differ between the groups, with similar estimations for the Mantel-Haenszel (OR = 1.34, 95% CI 0.95 to 1.89, χ^2^ = 3.06, p = 0.08) (Supplementary Figure S2) and Peto methods (OR = 1.37, 95% CI 0.96 to 1.95, χ^2^ = 3.06, p = 0.08).

#### VR-enhanced therapy compared to an active condition at post-treatment and follow-up

**Figure 4:**
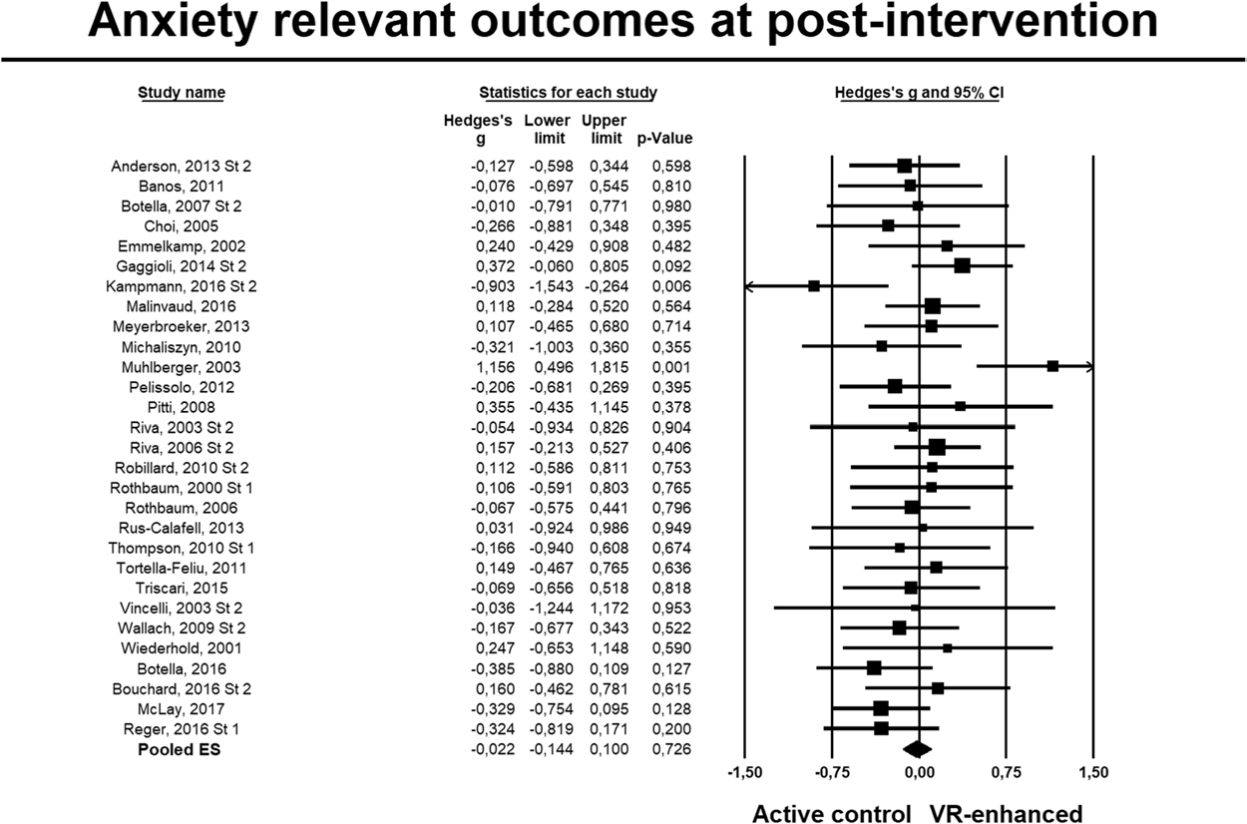
Forest plot: Standardized mean differences post-test for VR-enhanced therapy versus non-VR active psychological treatments for anxiety outcomes.

For anxiety (Fig. 4), twenty-nine RCTs were pooled, *g* = −0.02, 95% CI −0.14 to 0.10, with low heterogeneity (*I*^2^ = 20%, 95% CI 0 to 50). Analyses restricted to trials with participants with an anxiety disorder (23 comparisons) also resulted in non-significant effects (albeit slightly more favorable to the non-VR interventions), *g* = −0.10, 95% CI −0.24 to 0.04, with similar heterogeneity estimates, *I*^2^ = 26%, 95% CI 0 to 55. Results remained comparable after excluding two potential outliers, *g* = −0.02, 95% CI −0.13 to 0.08, *I*^2^ = 0%, and in analyses limited to trials with at least 25 participants randomized per arm, *g* = −0.05, 95% CI −0.19 to 0.07, *I*^2^ = 1% (Table 4).

**Table 4:** VR-enhanced therapy vs. active condition contrast, post-treatment.

Anxiety symptoms		N	g^a^	95% CI	*I* ^2^	I^2^ 95% CI	NNT	p^b^
All studies		29	−0.02	−0.14 to 0.10	20	0 to 50	83.33	
Outliers excluded^c^		27	−0.02	−0.13 to 0.08	0	0 to 43	83.33	
Only studies with >25 randomized per arm		12	−0.05	−0.19 to 0.07	1	0 to 59	35.71	
Only studies involving anxiety disorders		23	−0.10	−0.24 to 0.04	26	0 to 55	17.86	
**Subgroup analyses** ^**d**^								
Country	N. America	9	−0.12	−0.31 to 0.06	0	0 to 65	14.71	0.198
	EU	18	0.04	−0.13 to 0.23	39	0 to 65	45.45	
VR program author	N	8	0.09	−0.21 to 0.40	54	0 to 79	20.00	0.372
among author pool^e^	Y	20	−0.05	−0.18 to 0.07	0	0 to 48	35.71	
Recruitment^f^	Army	2	−0.32	−0.64 to −0.005	0	N/A^i^	5.56	
	Clinic	7	0.03	−0.17 to 0.23	0	0 to 71	62.50	0.159
	Community	17	0.001	−0.19 to 0.19	43	0 to 68	1772.4	
Control group	CBT	18	0.03	−0.09 to 0.16	0	0 to 50	62.50	0.120
	IE	6	−0.16	−0.41 to 0.08	0	0 to 75	11.11	
	IVE	4	−0.35	−0.78 to 0.07	49	0 to 83	5.10	
Experimental	VRCBT	17	0.09	−0.04 to 0.24	3	0 to 53	20.00	*0.016*
intervention	VRE	12	−0.18	−0.35 to −0.006	11	0 to 50	9.80	
Type of anxiety disorder	Flight anxiety	7	0.21	−0.12 to 0.54	41	0 to 75	8.47	0.206
	Panic disorder	6	−0.05	−0.32 to 0.21	0	0 to 75	35.71	
	PTSD	2	−0.32	−0.64 to −0.005	0	N/A	5.56	
	Social anxiety	5	−0.18	−0.52 to 0.15	41	0 to 78	9.80	
	Specific phobia	3	−0.19	−0.57 to 0.17	14	0 to 91	9.43	
Incomplete outcome data RoB^j^	High/Unclear	20	0.02	−0.11 to 0.15	5	0 to 50	83.33	0.326
	Low	9	−0.12	−0.36 to 0.12	43	0 to 74	14.71	
**Depressive symptoms**								
All studies		13	0.004	−0.20 to 0.21	26	0 to 62	443.11	
Outliers excluded^g^		12	0.07	−0.10 to 0.25	0	0 to 58	25.00	
Only studies with >25 randomized participants per arm		4	−0.03	−0.27 to 0.20	0	0 to 85	62.5	
**Subgroup analyses**								
Country	N. America	3	0.14	−0.19 to 0.48	0	0 to 90	12.82	0.410
	EU	9	−0.04	−0.32 to 0.24	40	0 to 72	45.45	
VR program author	N	2	0.03	−0.37 to 0.43	0	N/A	62.50	0.901
among author pool	Y	11	−0.001	−0.24 to 0.24	37	0 to 69	1772.4	
Recruitment^h^	Clinic	6	−0.01	−0.25 to 0.23	0	0 to 75	166.67	0.769
	Community	4	−0.12	−0.79 to 0.55	76	32 to 91	14.71	
Control group	CBT	10	0.08	−0.10 to 0.28	0	0 to 62	21.74	0.777
	IE	2	0.02	−0.39 to 0.43	0	N/A	83.33	
Experimental	VRCBT	8	0.17	−0.07 to 0.43	0	0 to 68	10.42	0.126
intervention	VRE	5	−0.18	−0.57 to 0.20	62	0 to 86	9.80	
Incomplete outcome data RoB	High/Unclear	9	0.08	−0.11 to 0.29	0	0 to 65	21.74	0.308
	Low	4	−0.25	−0.88 to 0.37	70	15 to 90	7.14	

^a^All results are reported with Hedges’ *g*, using a random effects model. Negative effect indicates superiority of the active interventions over the VR-enhanced therapies.

^b^The p levels in this column indicate whether the difference between the ESs in the subgroups is significant. (significant results are marked with italic).

^c^Outliers: Kampmann, 2016 St.2; Muhlberger, 2003.

^d^Subgroup analysis were conducted using a mixed effects model. Only subgroups with at least 2 studies were included.

^e^One study (Wiederhold, 2001) did not contain information about this moderator.

^f^Two studies (Meyerbroeker, 2013, Robillard, 2010 St2) did not contain information about this moderator.

^g^Outliers: Kampmann, 2016 St.2.

^h^One study (Robillard, 2010 St.2) did not contain information about this moderator.

^i^Confidence intervals around *I*^*2*^ cannot be calculated if there are less than 3 groups.

^j^RoB: Risk of Bias.

For *depression*, thirteen RCTs were aggregated, *g* = 0.004, 95% CI: −0.20 to 0.21, with low heterogeneity (*I*^2^ = 26%, 95% CI0 to 62). Exclusion of one outlier led to similar estimations, *g* = 0.07, 95% CI −0.10 to 0.25, *I*^2^ = 0%, as did analyses excluding small N studies, *g* = −0.03, 95% CI −0.27 to 0.20, *I*^2^ = 0%.

*Follow-up* anxiety outcomes were reported in 15 RCTs, g = −0.07, 95% CI −0.28 to 0.13, with moderate heterogeneity (*I*^2^ = 40%, 95% CI 0 to 75). Results were similar with the exclusion of one outlier, *g* = −0.02, 95% CI −0.19 to 0.14, *I*^2^ = 8%. Depressive symptoms at follow-up were reported in 5 RCTs, *g* = −0.19, 95% CI −0.62 to 0.23, with moderate heterogeneity (*I*^*2*^ = 57%).

Eighteen trials reported non-zero *drop-outs* in at least one group and ten trials reported zero drop-outs in both groups (Supplementary Table S2). Drop-out rates did not significantly differ between the groups, with similar results for the Mantel-Haenszel (OR = 1.05, 95% CI 0.77 to 1.43, χ^2^ = 14.06, *p* = 0.66) (Supplementary Figure S3) and Peto methods (OR = 1.05, 95% CI 0.77 to 1.43, χ^2^ = 0.12, *p* = 0.72).

#### Subgroup and meta-regression analyses

Recruitment setting was a significant moderator for the comparison between VR-enhanced interventions and control (*p* = 0.02) for anxiety, with the smallest ESs for recruitment from army settings and the highest for recruitment from a clinic. The type of anxiety disorder was also a significant moderator (*p* < 0.01), but this result is most likely affected by the high heterogeneity present within some of the small subgroups, as shown by the very large confidence intervals around *I*^*2*^. Effects were very high for specific phobia (3 trials, *g* = 1.79, 95% CI 0.64 to 2.94) and panic disorder, though the latter was only studied in 2 trials. Effects were also high for flight anxiety (3 trials, *g* = 0.82, 95% CI 0.42 to 1.22). Effects were small for PTSD (4 trials, *g* = 0.39, 95% CI 0.04 to 0.74), and moderate for social anxiety (5 trials, *g* = 0.67, 95% CI 0.25 to 1.09). In the comparison with other active therapies, the type of VR intervention (VRE vs VR CBT) was a significant moderator (*p* = 0.02) for anxiety outcomes. In the subgroup (12 comparisons) where the VR-enhanced therapy was VRE, the non-VR intervention was slightly more effective (*g* = −0.18, 95% CI −0.35 to −0.006). In this subgroup, the non-VR intervention consisted of imaginal exposure (6 comparisons), CBT (2 comparisons) and *in vivo* exposure (4 comparisons) (Tables 3 and 4).

Univariate meta-regression indicated significant negative relationships between publication year and both anxiety (slope = −0.06, 95% CI: −0.09 to −0.03) and depression ESs (slope = −0.10, 95% CI: −0.18 to −0.02) in comparison with control conditions, which were maintained in sensitivity analyses excluding outliers. The number of elements of interaction with the virtual environment was positively associated with anxiety outcomes (slope = 0.22, 95% CI: 0.01 to 0.42), but this result did not survive in a sensitivity analysis excluding outliers. For the contrast with other active conditions, publication year, mean age and respectively RoB score were significantly related to anxiety ESs, but only the relationship with age (slope = 0.02, 95% CI: 0.006 to 0.04) survived in analyses excluding outliers.

#### Small study effects and publication bias

**Figure 5:**
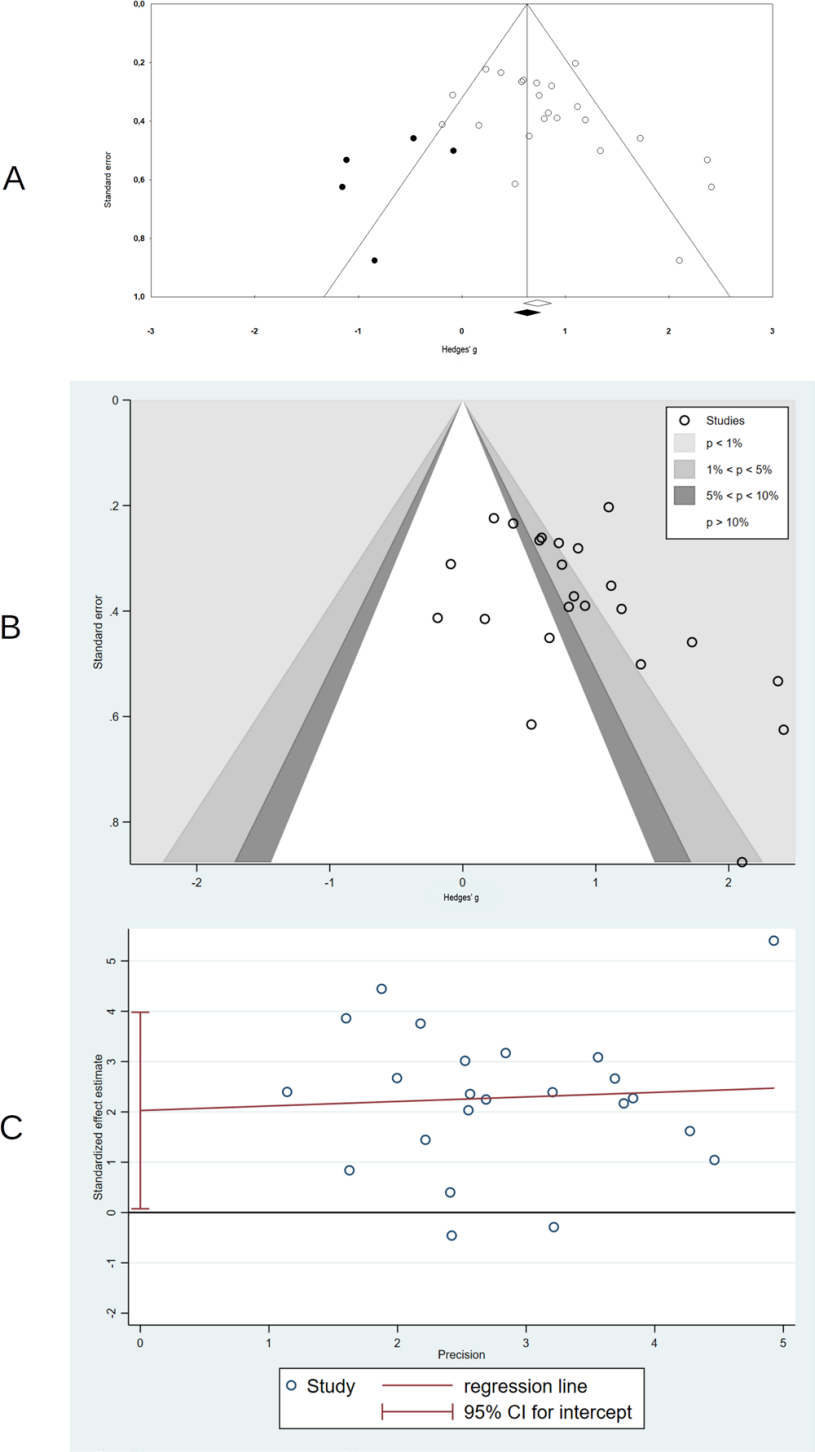
Funnel plots for comparison between VR-enhanced therapy and control conditions for anxiety outcomes: (**A**) Trim and fill adjusted (white circles, observed studies; black circles, imputed studies); (**B**) Contour-enhanced funnel plot; (**C**) Galbraith plot.

Visual inspection pointed to an asymmetrical funnel for both anxiety and depression. Contour enhanced funnel plots showed that for anxiety (Fig. 5), most of the studies with higher standard errors had results overcoming conventional statistical threshold of *p* < 0.05, with a considerable proportion of these even significant at the more conservative threshold of *p* < 0.01. Results were similar for depression (Figure S4), though the number of ESs was much smaller. Egger’s regression intercept test was statistically significant for both anxiety (intercept = 2.03, 95% CI 0.07 to 3.98, *p* = 0.04) and depression outcomes (intercept = 3.24, 95% CI 0.10 to 6.39, *p* = 0.04). Galbraith plots for anxiety (Fig. 5) evidenced the same pattern, as studies with low precision (i.e., inverse of the standard error) did not scatter randomly around the regression line, with most of them having effect estimations benefiting the VR intervention. For depression (Supplementary Figure S4) the pattern was inconclusive, probably due to the small number of studies. Finally, the Duval and Tweedie’s trim and fill procedure also pointed to small study effects for anxiety and depression. For anxiety, adjustment for potentially missing studies (n = 5), was associated with the ES decreasing from 0.79 to 0.62, whereas for depression (n = 3), it rendered the pooled ES non-significant. There was reduced indication of small study effects or publication bias for the comparison with other active treatments, with Egger’s test non-significant and no adjustment for missing studies, except for depression.

For drop-out rates, the Harbord test did not indicate small study effects (coeff = 0.16, 95% CI −1.92 to 2.24, *p* = 0.87). However, it is important to note this analysis may be biased, as it excluded studies with zero drop-out counts in both arms, which were also some of the smaller N studies (Supplementary Table S2).

## Discussion

In the reported meta-analysis, we showed moderate to large effects of VR interventions compared to control conditions (e.g., waitlist, placebo, relaxation, treatment as usual), for anxiety and depression outcomes. The number of studies with follow-up evaluations was too small for a meaningful ES estimation. There was moderate to high heterogeneity and a number of studies with extreme values. Most studies had a small number of participants and there was substantial evidence of small study effects for anxiety outcomes, pointing to potential publication bias. The limited number of studies reporting on depression outcomes precluded us from drawing a meaningful conclusion about small study effects. Adjustment for funnel plot asymmetry, as well as sensitivity analyses excluding outliers or restricted to studies with a moderate number of randomized participants per arm reduced the pooled ES for anxiety, though it still remained moderate to large. Only 7 trials that reported on anxiety outcomes had randomized at least 25 participants in each arm. The persistent evidence of small study effects, as well as the significant heterogeneity, y casts doubts over the reliability of the large effects observed for anxiety^25,37,38^. Heterogeneity continued to remain moderate with large confidence intervals even when extreme values were excluded, showing it was not simply the by-product of a few trials. Two thirds of the studies used waitlist controls, and effect sizes were large in waitlist comparisons. Use of waitlist controls might inadvertently and artificially inflate effect sizes for both anxiety and depression outcomes^39,40^.

Conversely, compared with established active interventions, effect sizes were non-significant for both anxiety and depression outcomes, at post-test and follow-up. Heterogeneity was small to moderate and there was limited evidence of funnel plot asymmetry or small study effects. Sensitivity analyses excluding outliers or restricted to studies with at least 25 participants randomized in each arm produced similar estimations. There were more trials in the latter category (12) than in the comparison with control conditions (7), but these were still a minority. All but one of the trials were powered to test superiority, not equivalence or non-inferiority^41^, so it would be premature to construe our findings as proof of equivalent effects. Most frequently employed non-VR active interventions were IVE and CBT, both shown to be effective for anxiety and depression, thereby potentially difficult to outperform.

VR-enhanced interventions did not improve attrition, producing similar drop-out rates with control conditions and other active interventions. These findings contradict previous speculation of possible comparative benefit^1,5,8^. However, most trials were small and many reported zero drop-outs, sometimes in both arms, so the stability of this result needs to be considered with caution. We were not able to evidence small study effects for analyses on attrition, but this result is most likely biased by the fact studies with zero counts in both arms were excluded and many of these were also small studies.

The vast majority of RCTs of VR interventions had high or uncertain risk of bias across domains. Two previous meta-analyses^6,7^ examined bias using combinations of instruments, which included aspects not linked to any type of trial bias (e.g., training for providers), potentially obfuscating distorting effects. In contrast, we used the Cochrane Risk of Bias tool^11^, which evaluates domains likely to distort outcomes. Only four trials could be rated as low RoB on all domains considered, preventing us from reliably assessing the relationship between overall trial risk of bias and outcomes. The only RoB domain where most trials reported information was incomplete outcome data. Almost two thirds of the studies were rated as high risk of attrition bias, again questioning the reliability of the ES estimations, as exclusion of participants from RCT analyses was shown to distort outcomes^42,43^. In exploratory subgroup analysis, we did not find differences between studies with high/uncertain versus low RoB for incomplete outcome reporting, though the number of studies with low RoB was small, particularly in comparisons with control (7). It is possible previous assessments concluding no relationship between trial risk of bias and ESs might have been too optimistic.

Though the presence the developers of VR interventions among the author pool was not significantly associated with changes in the magnitude of the effects, it is worth underscoring the vast majority of trials did involve such a developer. For instance, for the comparison with control conditions, only five anxiety effect sizes came from independent studies, and 17 from trials involving the developer. As such, it is possible that the insufficient variability in our sample of included trials prevented us from detecting more subtle differences. Moreover, we only examined whether one of the authors had also developed the VR treatment program used, not any potential commercial involvements with VR companies, which could arguably represent a more direct conflict of interest. However, since most articles did not report this information, we could not examine it systematically.

We identified few moderators, owing to the fact most subgroups were small and affected by high heterogeneity within the group. Recruitment setting seemed to have an influence on ESs in comparisons between VR-enhanced and control conditions, with smaller effects for recruitment from army settings, but this may also be a spurious result since some of the subgroups contained a very limited number of studies. Type of anxiety diagnosis also appeared to be a significant moderator, with high effects for specific phobia and flight anxiety, and moderate or small effects for social anxiety and PTSD. It is likely that this is at least partly a spurious result, given subgroups were small and heterogeneity was high in all of them. The type of active comparison intervention used appeared to matter, with VR-enhanced exposure having slightly smaller effects than non-VR interventions. Again, the number of studies was small and this relationship could have also been confounded by other variables, such as the type of problem for which the therapy was used.

It was speculated^1^ that improved engagement with the virtual environment, as measured by immersion or a sense of presence, could play an important role in the effectiveness of VR. Only a modest number of trials measured immersion and presence explicitly. Even in those that did, most did not analyze these variables in relationship to treatment outcomes or found no association. We showed that the number of elements employed by the VR technology, a crude indicator of interaction, was positively related with anxiety outcomes in comparisons with passive, but not active, treatments. However, this result did not survive sensitivity analyses and could be an artefactual finding. But even for visual stimulation, though one might assume that more recent studies use very sophisticated technology, instead of stereoscopic simulations not intended for VR use, we saw no evidence to this effect. For example a 2017 trial^44^ relied on the same technology as similar trials from 2013^45^ and even 2005^46^.

Publication year was consistently negatively associated to outcomes, though reasons for this trend remained unclear. A rise in larger or lower risk of bias trials seems unlikely given we observed few such trials. The apparent decrease in effectiveness with the passing of time might also be a by-product of the early use of pilot, low powered studies where only large effects can overcome the significance threshold, a strong initial publication bias for positive findings, as well as time lag bias, whereby studies with positive results are published first and dominate the field, until the negative, but equally important, studies get published^22,47^. Previous meta-analyses of RCTs of VR interventions either did not consider publication bias at all^4,6^, or reported optimistic estimations^7^, based on the fail-safe N, whose use is discouraged for being unreliable and misleading^22^. We used a range of methods to assess funnel plot asymmetry, all of which corroborated that small studies were numerous, mostly significant and overestimated effects for comparisons with control conditions. Publication bias for positive findings, probably more prominent in the early years of studying VR interventions, is one likely cause of small study effects. We conjecture it is most likely present in the literature of VR interventions for anxiety, where most trials are concentrated.

There are several limitations to our meta-analysis. There was a high degree of heterogeneity, particularly in comparisons with control conditions. This was accompanied by very large confidence intervals around *I*^2^, even for the comparisons where heterogeneity estimates were smaller. Residual heterogeneity persisted even after sensitivity analyses were conducted, or potential moderators explored. NNTs can be useful as an ancillary clinical ES measure, but there is disagreement regarding the most adequate calculation method^48^, and concerns over their potential to mislead, particularly when resulting from meta-analyses, as baseline risk can vary substantially between trials^49^. Many of the subgroup analyses were underpowered and we were able to identify few moderators. We could not calculate effect sizes for three trials where the report did not contain enough information and the original authors did not provide the data. However, given their size and the total number of included trials, their exclusion is unlikely to have influenced estimations.

## Conclusions

From the standpoint of dissemination and implementation, our results leave several open questions. Virtual reality enhanced interventions had moderate to large effects compared to control conditions, though these effects were likely inflated by several factors in the design and implementation of the trials. We could find few difference with other active interventions. These might be construed as evidence VR-enhanced interventions could be added to the armamentarium, as another effective choice available to clinicians and patients.

However, other key aspects remain unclear. Though it would be intuitive to consider VR-enhanced interventions as more cost-effective than traditional anxiety treatments, notably *in vivo* exposure, research substantiating this claim is missing. Moreover, it might hinge on the specific disorder targeted. For instance, for flight anxiety it may seem evident that it would be more cost-effective to conduct VR-enhanced exposure than buy a plane ticket for *in vivo* exposure. Conversely, for height anxiety, it could be more cost-effective to scale a flight of stairs with a patient, than to purchase a HMD system and pay for the software development of a fully immersive VR environment. Nonetheless, this kind of tailored, immersive and sophisticated technology does not seem to be used much, even in recent trials, further complicating a realistic calculation of cost-effectiveness. One might also argue VR-enhanced interventions might be particularly suitable for disorders where other active interventions have been less effective. Nonetheless, in the case of one such disorder- post-traumatic stress disorder- two recent trials^36,50^ failed to find additional benefits for VR interventions over non-VR treatments such as prolonged exposure, both in terms of primary outcome, as well as drop-out rates, with follow-up results actually better for the non-VR intervention.

Most importantly, many existent trials are poorly reported and exposed to bias. The effort to move forward should primarily focus on elevating the quality of VR trials. Larger trials minimizing risk of bias by prospective registration and transparent and complete reporting, as well as using credible control group, are necessary. A recent ongoing trial described in a published protocol is one such example^51^. Trials should also report cost-effectiveness analyses in an attempt to clarify whether and under which conditions are VR-enhanced treatments cost-effective. Finally, they should include an evaluation of the participants’ engagement with the VR environment, so as to clarify how immersive and sophisticated the system needs to be to support improved outcomes. Moreover, given the predominance of trials conducted by developers of VR treatments, independently conducted trials are also critical. It is essential that negative results are afforded journal space in order to tackle potential publication bias.

## Electronic supplementary material

Supplementary information
