# Supplementary material for: The effectiveness of virtual reality based interventions for symptoms of anxiety and depression: A meta-analysis
Source: Sci Rep. 2018 Jul 9;8:10323. doi: 10.1038/s41598-018-28113-6 (PMC6037699; doi:10.1038/s41598-018-28113-6)

The effectiveness of virtual reality based interventions for symptoms of anxiety and depression: A meta-analysis

Liviu A. Fodor[a,b], Carmen D. Coteț[c], Pim Cuijpers[d], Ștefan Szamoskozi[e], Daniel David[c,f], Ioana A. Cristea[c,g,*]

[a] International Institute for The Advanced Studies of Psychotherapy and Applied Mental Health, Babeș-Bolyai University, Cluj-Napoca, Romania

[b] Evidence Based Psychological Assessment and Interventions Doctoral School, Babeș-Bolyai University, Cluj-Napoca, Romania

[c] Department of Clinical Psychology and Psychotherapy, Babeş-Bolyai University, Republicii Street 37, 400015, Cluj-Napoca, Romania

[d] Department of Clinical, Neuro and Developmental Psychology, Vrije Universiteit, Amsterdam, the Netherlands; Amsterdam Public Health Research Institute, Vrije Universiteit, Amsterdam, the Netherlands

[e] Department of Applied Psychology, December 21 1989 Street 128, Babeș-Bolyai University, Cluj-Napoca

[f] Department of Oncological Sciences, Icahn School of Medicine at Mount Sinai, New York, USA

[g] Meta-Research Innovation Center at Stanford, Stanford University, Stanford, California

* Corresponding author at: Department of Clinical Psychology and Psychotherapy, Babeş-Bolyai University, Republicii Street 37, 400015 Cluj-Napoca, Romania

E-mail address: ioana.cristea@ubbcluj.ro (Cristea, I.A.)

Supplementary Method: Complete search string

Search string Pubmed: "virtual reality"[Title/Abstract] AND ("therapy"[Title/Abstract] OR "exposure"[Title/Abstract] OR "intervention"[Title/Abstract] OR "treatment"[Title/Abstract])

Limits: randomized trials

Search date: August 10th, 2016

Hits: 229

Supplementary Results: List of included studies

Anderson, P. L., Price, M., Edwards, S. M., Obasaju, M. A., Schmertz, S. K., Zimand, E., & Calamaras, M. R. (2013). Virtual reality exposure therapy for social anxiety disorder: a randomized controlled trial. Journal of Consulting and Clinical Psychology, 81(5), 751–760. https://doi.org/10.1037/a0033559

Baños, R. M., Guillen, V., Quero, S., García-Palacios, A., Alcaniz, M., & Botella, C. (2011). A virtual reality system for the treatment of stress-related disorders: A preliminary analysis of efficacy compared to a standard cognitive behavioral program. International Journal of Human-Computer Studies, 69(9), 602–613. https://doi.org/10.1016/j.ijhcs.2011.06.002

Botella, C., García-Palacios, A., Villa, H., Baños, R. M., Quero, S., Alcañiz, M., & Riva, G. (2007). Virtual reality exposure in the treatment of panic disorder and agoraphobia: A controlled study. Clinical Psychology & Psychotherapy, 14(3), 164–175. https://doi.org/10.1002/cpp.524

Botella, C., Pérez-Ara, M. Á., Bretón-López, J., Quero, S., García-Palacios, A., & Baños, R. M. (2016). In Vivo versus Augmented Reality Exposure in the Treatment of Small Animal Phobia: A Randomized Controlled Trial. PLOS ONE, 11(2), e0148237. https://doi.org/10.1371/journal.pone.0148237

Bouchard, S., Dumoulin, S., Robillard, G., Guitard, T., Klinger, É., Forget, H., … Roucaut, F. X. (2016). Virtual reality compared with in vivo exposure in the treatment of social anxiety disorder: a three-arm randomised controlled trial. The British Journal of Psychiatry, bjp.bp.116.184234. https://doi.org/10.1192/bjp.bp.116.184234

Choi, Y.-H., Vincelli, F., Riva, G., Wiederhold, B. K., Lee, J.-H., & Park, K.-H. (2005). Effects of group experiential cognitive therapy for the treatment of panic disorder with agoraphobia. Cyberpsychology & Behavior: The Impact of the Internet, Multimedia and Virtual Reality on Behavior and Society, 8(4), 387–393. https://doi.org/10.1089/cpb.2005.8.387

Emmelkamp, P. M. G., Krijn, M., Hulsbosch, A. M., de Vries, S., Schuemie, M. J., & van der Mast, C. A. P. G. (2002). Virtual reality treatment versus exposure in vivo: a comparative evaluation in acrophobia. Behaviour Research and Therapy, 40(5), 509–516. https://doi.org/10.1016/S0005-7967(01)00023-7

Gaggioli, A., Pallavicini, F., Morganti, L., Serino, S., Scaratti, C., Briguglio, M., … Riva, G. (2014). Experiential virtual scenarios with real-time monitoring (interreality) for the management of psychological stress: a block randomized controlled trial. Journal of Medical Internet Research, 16(7), e167. https://doi.org/10.2196/jmir.3235

Garcia-Palacios, A., Hoffman, H., Carlin, A., Furness, T. A., & Botella, C. (2002). Virtual reality in the treatment of spider phobia: a controlled study. Behaviour Research and Therapy, 40(9), 983–993.

Kampmann, I. L., Emmelkamp, P. M. G., Hartanto, D., Brinkman, W.-P., Zijlstra, B. J. H., & Morina, N. (2016). Exposure to virtual social interactions in the treatment of social anxiety disorder: A randomized controlled trial. Behaviour Research and Therapy, 77, 147–156. https://doi.org/10.1016/j.brat.2015.12.016

Krijn, M., Emmelkamp, P. M. G., Biemond, R., de Wilde de Ligny, C., Schuemie, M. J., & van der Mast, C. A. P. G. (2004). Treatment of acrophobia in virtual reality: the role of immersion and presence. Behaviour Research and Therapy, 42(2), 229–239. https://doi.org/10.1016/S0005-7967(03)00139-6

Lau, W.-C., Choi, K.-S., & Chung, W.-Y. (2010). A virtual psychiatric ward for orientating patients admitted for the first time. Cyberpsychology, Behavior and Social Networking, 13(6), 637–648. https://doi.org/10.1089/cyber.2009.0107

Malinvaud, D., Londero, A., Niarra, R., Peignard, P., Warusfel, O., Viaud-Delmon, I., … Bonfils, P. (2016). Auditory and visual 3D virtual reality therapy as a new treatment for chronic subjective tinnitus: Results of a randomized controlled trial. Hearing Research, 333, 127–135. https://doi.org/10.1016/j.heares.2015.12.023

Maltby, N., Kirsch, I., Mayers, M., & Allen, G. J. (2002). Virtual reality exposure therapy for the treatment of fear of flying: a controlled investigation. Journal of Consulting and Clinical Psychology, 70(5), 1112–1118.

McLay, R. N., Baird, A., Webb-Murphy, J., Deal, W., Tran, L., Anson, H., … Johnston, S. (2017). A Randomized, Head-to-Head Study of Virtual Reality Exposure Therapy for Posttraumatic Stress Disorder. Cyberpsychology, Behavior and Social Networking, 20(4), 218–224. https://doi.org/10.1089/cyber.2016.0554

McLay, R. N., Wood, D. P., Webb-Murphy, J. A., Spira, J. L., Wiederhold, M. D., Pyne, J. M., & Wiederhold, B. K. (2011). A randomized, controlled trial of virtual reality-graded exposure

therapy for post-traumatic stress disorder in active duty service members with combat-related post-traumatic stress disorder. Cyberpsychology, Behavior and Social Networking, 14(4), 223–229. https://doi.org/10.1089/cyber.2011.0003

Meyerbroeker, K., Morina, N., Kerkhof, G. A., & Emmelkamp, P. M. G. (2013). Virtual reality exposure therapy does not provide any additional value in agoraphobic patients: a randomized controlled trial. Psychotherapy and Psychosomatics, 82(3), 170–176. https://doi.org/10.1159/000342715

Michaliszyn, D., Marchand, A., Bouchard, S., Martel, M.-O., & Poirier-Bisson, J. (2010). A randomized, controlled clinical trial of in virtuo and in vivo exposure for spider phobia. Cyberpsychology, Behavior and Social Networking, 13(6), 689–695. https://doi.org/10.1089/cyber.2009.0277

Miyahira, S. D., Folen, R. A., Hoffman, H. G., Garcia-Palacios, A., Spira, J. L., & Kawasaki, M. (2012). The effectiveness of VR exposure therapy for PTSD in returning warfighters. Studies in Health Technology and Informatics, 181, 128–132.

Mühlberger, A., Herrmann, M. J., Wiedemann, G. C., Ellgring, H., & Pauli, P. (2001). Repeated exposure of flight phobics to flights in virtual reality. Behaviour Research and Therapy, 39(9), 1033–1050.

Mühlberger, A., Wiedemann, G., & Pauli, P. (2003). Efficacy of a one-session virtual reality exposure treatment for fear of flying. Psychotherapy Research: Journal of the Society for Psychotherapy Research, 13(3), 323–336. https://doi.org/10.1093/ptr/kpg030

Pelissolo, A., Zaoui, M., Aguayo, G., Sai, N., Yao, Roche, S., … Cottraux, J. (2012). Virtual reality exposure therapy versus cognitive behavior therapy for panic disorder with agoraphobia: A randomized comparison study. ResearchGate, 5(1). Retrieved from https://www.researchgate.net/publication/233997242_Virtual_reality_exposure_therapy_versus_cognitive_behavior_therapy_for_panic_disorder_with_agoraphobia_A_randomized_comparison_study

Pitti, C., Peñate, W., de la Fuente, J., Bethencourt, J., Acosta, L., Villaverde, M., & Gracia, R. (2008). [Agoraphobia: combined treatment and virtual reality. Preliminary results]. Actas Espanolas De Psiquiatria, 36(2), 94–101.

Ready, D. J., Gerardi, R. J., Backscheider, A. G., Mascaro, N., & Rothbaum, B. O. (2010). Comparing virtual reality exposure therapy to present-centered therapy with 11 U.S.

Vietnam veterans with PTSD. Cyberpsychology, Behavior and Social Networking, 13(1), 49–54.

Reger, G. M., Koenen-Woods, P., Zetocha, K., Smolenski, D. J., Holloway, K. M., Rothbaum, B. O., … Gahm, G. A. (2016). Randomized controlled trial of prolonged exposure using imaginal exposure vs. virtual reality exposure in active duty soldiers with deployment-related posttraumatic stress disorder (PTSD). Journal of Consulting and Clinical Psychology, 84(11), 946–959. https://doi.org/10.1037/ccp0000134

Riva, G., Bacchetta, M., Cesa, G., Conti, S., & Molinari, E. (2003). Six-month follow-up of in-patient experiential cognitive therapy for binge eating disorders. Cyberpsychology & Behavior: The Impact of the Internet, Multimedia and Virtual Reality on Behavior and Society, 6(3), 251–258. https://doi.org/10.1089/109493103322011533

Riva, G., Bacchetta, M., Cesa, G., Conti, S., Castelnuovo, G., Mantovani, F., & Molinari, E. (2006). Is severe obesity a form of addiction? Rationale, clinical approach, and controlled clinical trial. Cyberpsychology & Behavior: The Impact of the Internet, Multimedia and Virtual Reality on Behavior and Society, 9(4), 457–479. https://doi.org/10.1089/cpb.2006.9.457

Robillard, G., Bouchard, S., Dumoulin, S., Guitard, T., & Klinger, E. (2010). Using virtual humans to alleviate social anxiety: preliminary report from a comparative outcome study. Studies in Health Technology and Informatics, 154, 57–60.

Rothbaum, B. O., Anderson, P., Zimand, E., Hodges, L., Lang, D., & Wilson, J. (2006). Virtual reality exposure therapy and standard (in vivo) exposure therapy in the treatment of fear of flying. Behavior Therapy, 37(1), 80–90. https://doi.org/10.1016/j.beth.2005.04.004

Rothbaum, B. O., Hodges, L. F., Kooper, R., Opdyke, D., Williford, J. S., & North, M. (1995). Effectiveness of computer-generated (virtual reality) graded exposure in the treatment of acrophobia. The American Journal of Psychiatry, 152(4), 626–628. https://doi.org/10.1176/ajp.152.4.626

Rothbaum, B. O., Hodges, L., Smith, S., Lee, J. H., & Price, L. (2000). A controlled study of virtual reality exposure therapy for the fear of flying. Journal of Consulting and Clinical Psychology, 68(6), 1020–1026.

Rus-Calafell, M., Gutiérrez-Maldonado, J., Botella, C., & Baños, R. M. (2013). Virtual reality exposure and imaginal exposure in the treatment of fear of flying: a pilot study. Behavior Modification, 37(4), 568–590. https://doi.org/10.1177/0145445513482969

Stetz, M. C., Kaloi-Chen, J. Y., Turner, D. D., Bouchard, S., Riva, G., & Wiederhold, B. K. (2011). The effectiveness of technology-enhanced relaxation techniques for military medical warriors. Military Medicine, 176(9), 1065–1070.

Thompson, T., Steffert, T., Steed, A., & Gruzelier, J. (2011). A randomized controlled trial of the effects of hypnosis with 3-D virtual reality animation on tiredness, mood, and salivary cortisol. The International Journal of Clinical and Experimental Hypnosis, 59(1), 122–142. https://doi.org/10.1080/00207144.2011.522917

Tortella-Feliu, M., Botella, C., Llabrés, J., Bretón-López, J. M., del Amo, A. R., Baños, R. M., & Gelabert, J. M. (2011). Virtual reality versus computer-aided exposure treatments for fear of flying. Behavior Modification, 35(1), 3–30. https://doi.org/10.1177/0145445510390801

Triscari, M. T., Faraci, P., Catalisano, D., D'Angelo, V., & Urso, V. (2015). Effectiveness of cognitive behavioral therapy integrated with systematic desensitization, cognitive behavioral therapy combined with eye movement desensitization and reprocessing therapy, and cognitive behavioral therapy combined with virtual reality exposure therapy methods in the treatment of flight anxiety: a randomized trial. Neuropsychiatric Disease and Treatment, 11, 2591–2598. https://doi.org/10.2147/NDT.S93401

Vincelli, F., Anolli, L., Bouchard, S., Wiederhold, B. K., Zurloni, V., & Riva, G. (2003). Experiential cognitive therapy in the treatment of panic disorders with agoraphobia: a controlled study. Cyberpsychology & Behavior: The Impact of the Internet, Multimedia and Virtual Reality on Behavior and Society, 6(3), 321–328. https://doi.org/10.1089/109493103322011632

Wallach, H. S., Safir, M. P., & Bar-Zvi, M. (2009). Virtual Reality Cognitive Behavior Therapy for Public Speaking Anxiety A Randomized Clinical Trial. Behavior Modification, 33(3), 314–338. https://doi.org/10.1177/0145445509331926

Wiederhold, B. K., Gevirtz, R. N., & Spira, J. L. (2001). Virtual reality exposure therapy vs. imagery desensitization therapy in the treatment of flying phobia. In G. Riva & C. Galimberti (Eds.), Towards cyberpsychology: Mind, cognition and society in the internet age (pp. 253–272). Amsterdam, Netherlands: IOS Press.

Supplementary Figure S1. Risk of bias summary: review authors' judgments about each risk of bias item for each included study

	Random sequence generation (selection bias)	Allocation concealment (selection bias)	Blinding of outcome assessment (detection bias)	Incomplete outcome data (attrition bias)
Anderson, 2013	+	+	+	+
Banos, 2011	?	?	+	-
Botella, 2007	?	+	+	?
Botella, 2016	+	+	?	+
Bouchard, 2016	+	+	+	+
Choi, 2005	?	?	+	?
Emmelkamp, 2002	?	?	+	-
Gaggioli, 2014	?	?	+	+
Garcia-Palacios, 2002	?	?	-	+
Kampmann, 2016	+	+	+	+
Krijn, 2004	?	?	+	-
Lau, 2010	?	-	+	?
Malinvaud, 2016	+	?	+	-
Maltby, 2002	?	?	+	-
McLay, 2011	?	+	+	-
McLay, 2017	?	+	+	-
Meyerbroeker, 2013	?	?	-	-
Michaliszyn, 2010	?	?	?	?
Miyahira, 2012	?	?	?	-
Muhlberger, 2001	?	?	+	-
Muhlberger, 2003	-	?	+	-
Pelissolo, 2012	?	?	?	-
Pitti, 2008	+	?	-	-
Ready, 2010	?	?	+	-
Reger, 2016	+	+	+	+
Riva, 2003	?	?	+	?
Riva, 2006	?	?	+	-
Robillard, 2010	?	?	+	?
Rothbaum, 1995	?	?	+	-
Rothbaum, 2000	?	?	+	-
Rothbaum, 2006	?	?	+	+
Rus-Calafell, 2013	?	?	?	?
Stetz, 2011	?	?	+	-
Thompson, 2010	+	?	+	?
Tortella-Feliu, 2011	?	?	+	+
Triscari, 2015	+	-	+	?
Vincelli, 2003	?	?	+	+
Wallach, 2009	-	?	+	-
Wiederhold, 2001	+	-	+	-

Supplementary Table S1. Tools for participant interaction with the virtual environment

Study	Mental health problem[a]	VR system (visual interaction)[b]	VR interaction tools	Measure of immersion/presence[c]	Number of tools
Anderson, 2013	Social Anxiety	HMD	visual feedback, sound, participant speeches	NR	3
Banos, 2011	Mixed disorder	VR room	visual feedback, sound, navigation via wireless pad	NR	4
Botella, 2007	PD + AG	HMD	visual feedback, sound, navigation, mouse, interoceptive stimulation (e.g., breathing difficulties, tunnel vision)	NR	5
Botella, 2016	Spider Phobia	HMD	visual feedback, augmented reality (3D spiders superimposed on real world elements)	NR	2
Bouchard, 2016	Social Anxiety	HMD	visual feedback, sound, navigation, mouse, participant speeches, sitting/standing posture	PQ; GPQ; relationship with treatment outcomes not reported	6
Choi, 2005	PD + AG	HMD	visual feedback, navigation, joystick	NR	3
Emmelkamp, 2002	Acrophobia	HMD	visual feedback, railing to hold on to	NR	2
Gaggioli, 2014	Stress	HMD	visual feedback, sound, navigation, joystick	NR	4

Garcia-Palacios, 2002	Spider Phobia	HMD	visual feedback, sound, simulated VR hand for touching the spiders, tactile feedback	NR	4
Kampmann, 2016	Social Anxiety	HMD	visual feedback, sound, participant conversation with VR characters	NR	3
Krijn, 2004	Acrophobia	HMD/CV	visual feedback	IPQ; Presence not correlated to treatment outcomes	1
Lau, 2010	Ward orientation	PC	visual feedback, sound, navigation, keyboard/mouse	NR	4
Malinvaud, 2016	Tinnitus	HMD	visual feedback, sound, navigation, interaction via trackers	NR	4
Maltby, 2002	Flight Anxiety	HMD	visual feedback, sound, navigation, interaction via joystick	NR	4
McLay, 2011	PTSD	HMD	visual feedback, sound, navigation, joystick	NR	4
McLay, 2017	PTSD	HMD	visual feedback, sound, navigation, joystick, vibration, smells	NR	6
Meyerbroeker, 2013	PD + AG	HMD/CV	visual feedback, sound	NR	2
Michaliszyn, 2010	Spider Phobia	HMD	visual feedback, sound, navigation via handheld wireless mouse	PQ-F; High mean presence	3
Miyahira, 2012	PTSD	HMD	visual feedback, sound, kinesthetic stimuli	NR	3

Muhlberger, 2001	Flight Anxiety	HMD	visual feedback, sound, motion, acceleration/deceleration simulation via motion base with 6 dof	NR	4
Muhlberger, 2003	Flight Anxiety	HMD	visual feedback, sound, motion, acceleration/deceleration simulation via motion base with 6 dof	NR	4
Pelissolo, 2012	PD + AG	HMD	visual feedback, unclear sound rendering, unclear navigation device	NR	Unclear
Pitti, 2008	PD + AG.	CV	visual feedback, unclear sound rendering, unclear navigation device	NR	Unclear
Ready, 2010	PTSD	HMD	visual feedback, sound, navigation via joystick	NR	3
Reger, 2016	PTSD	HMD	visual feedback, sound, navigation via joystick+ mock rifle, vibration simulation via platform with low bass speakers	NR	4
Riva, 2003	BED	HMD	visual feedback, unclear sound rendering device, navigation via joystick	NR	Unclear
Riva, 2006	Severe Obesity	HMD	visual feedback, unclear sound rendering device, navigation via joystick	NR	Unclear
Robillard, 2010	Social Anxiety	HMD	visual feedback, unclear sound rendering device, unclear on navigation device	NR	Unclear
Rothbaum, 1995	Acrophobia	HMD	visual feedback, unclear sound rendering device, platform with railing, virtual hand, tactile feedback	NR	4

Rothbaum, 2000	Flight Anxiety	HMD	visual feedback, sound, vibration simulated via chair-integrated subwoofer, airline seatbelt	NR	4
Rothbaum, 2006	Flight Anxiety	HMD	visual feedback, sound, vibration simulated via chair-integrated subwoofer, airline seatbelt	NR	4
Rus-Calafell, 2013	Flight Anxiety	HMD	visual feedback, sound, navigation via mouse	PRJQ; Presence not correlated to treatment effectiveness	3
Stetz, 2011	Stress	Screen	visual feedback, sound	UQO-PQ, low mean presence	2
Thompson, 2011	Tiredness/Mood	HMD	visual feedback, sound	*Ad-hoc* scale, relationship with treatment outcomes not reported	2
Tortella-Feliu, 2011	Flight Anxiety	HMD	visual feedback, sound, navigation via mouse	NR	3
Triscari, 2015	Flight Anxiety	N/R	unclear	NR	Unclear
Vincelli, 2003	PD + AG.	HMD	visual feedback, sound, navigation, joystick	NR	4
Wallach, 2009	Social Anxiety	HMD	visual feedback, sound, participant speeches	NR	3
Wiederhold, 2001	Flight Anxiety	HMD	visual feedback	NR	1

[a] PD = panic disorder; ED = eating disorder; BED = binge eating disorder; AG = agoraphobia; PTSD = post-traumatic stress disorder
[b] HMD = head-mounted display; CV = Cave-type system;
[c] NR = not reported; PQ = Presence Questionnaire; GPQ = Gatineau Presence Questionnaire; IPQ = Igroup Presence Questionnaire; PQ-F = Presence Questionnaire, French version; PRJQ = Presence and Reality Judgment Questionnaire; UQO-PQ = Université du Québec en Outaouais Presence Questionnaire

Supplementary Table S2. Study characteristics regarding drop-outs and remaining participants in each treatment arm

Study	N_{drop} VR[a]	N_{remain} VR[b]	N_{drop} P.C[c]	N_{remain} P.C.[d]	N_{drop} A.I.[e]	N_{remain} A.I.[f]
Anderson, 2013	8	32	9	19	15	33
Banos, 2011	6	19	///[h]	///	5	20
Botella, 2007	0	12	0	13	0	12
Botella, 2016	0	32	///	///	0	31
Bouchard, 2016	2	15	4	16	4	18
Choi, 2005	0	20	///	///	0	20
Emmelkamp, 2002	N.C.[g]	N.C.	N.C.	N.C.	N.C.	N.C.
Gaggioli, 2014	0	40	0	39	0	42
Garcia-Palacios, 2002	0	12	0	11	///	///
Kampmann, 2016	5	15	4	16	3	17
Krijn, 2004	N.C.	N.C.	N.C.	N.C.	N.C.	N.C.
Lau, 2010	0	27	0	27	///	///
Malinvaud, 2016	11	50	4	25	14	44
Maltby, 2002	2	18	0	23	///	///
McLay, 2011	0	10	1	9	///	///
McLay, 2017	7	36	///	///	0	38
Meyerbroeker, 2013	11	16	2	16	10	15
Michaliszyn, 2010	2	14	///	///	2	14
Miyahira, 2012	17	12	3	10	///	///
Muhlberger, 2001	0	15	2	13	///	///
Muhlberger, 2003	4	26	///	///	4	11
Pelissolo, 2012	10	33	12	20	10	34
Pitti, 2008	N.C.	N.C.	N.C.	N.C.	N.C.	N.C.
Ready, 2010	1	5	1	4	///	///
Reger, 2016	24	30	7	47	22	32
Riva, 2003	0	9	0	9	0	9
Riva, 2006	1	56	0	53	2	52
Robillard, 2010	0	14	0	15	0	16
Rothbaum, 1995	2	10	1	7	///	///
Rothbaum, 2000	3	12	0	15	1	14
Rothbaum, 2006	4	25	0	25	4	25
Rus-Calafell, 2013	0	7	///	///	0	8
Stetz, 2011	0	30	0	30	///	///
Thompson, 2010	0	12	0	11	0	12
Tortella-Feliu, 2011	2	17	///	///	2	18
Triscari, 2015	0	21	///	///	0	22

Vincelli, 2003	0	4	0	4	0	4
Wallach, 2009	6	28	15	18	3	42
Wiederhold, 2001	3	6	///	///	1	8

[a] Number of participants that dropped out in the VR-enhanced (VR) treatment arm

[b] Number of remaining participants in the VR-enhanced (VR) treatment arm

[c] Number of participants that dropped out in the Passive Control (P.C.) treatment arm

[d] Number of remaining participants in the Passive Control (P.C.) treatment arm

[e] Number of participants that dropped out in the Active intervention (A.I.) treatment arm

[f] Number of remaining participants in the Active intervention (A.I.) treatment arm

[g] N.C. = not conclusive (the number of participants that dropped out from each treatment arm could not be determined)

[h] /// = the study did not have the respective treatment arm

Supplementary Figure S2. Forest plot: Odds ratio post-test for VR-enhanced therapy versus control conditions at post-treatment for drop-outs

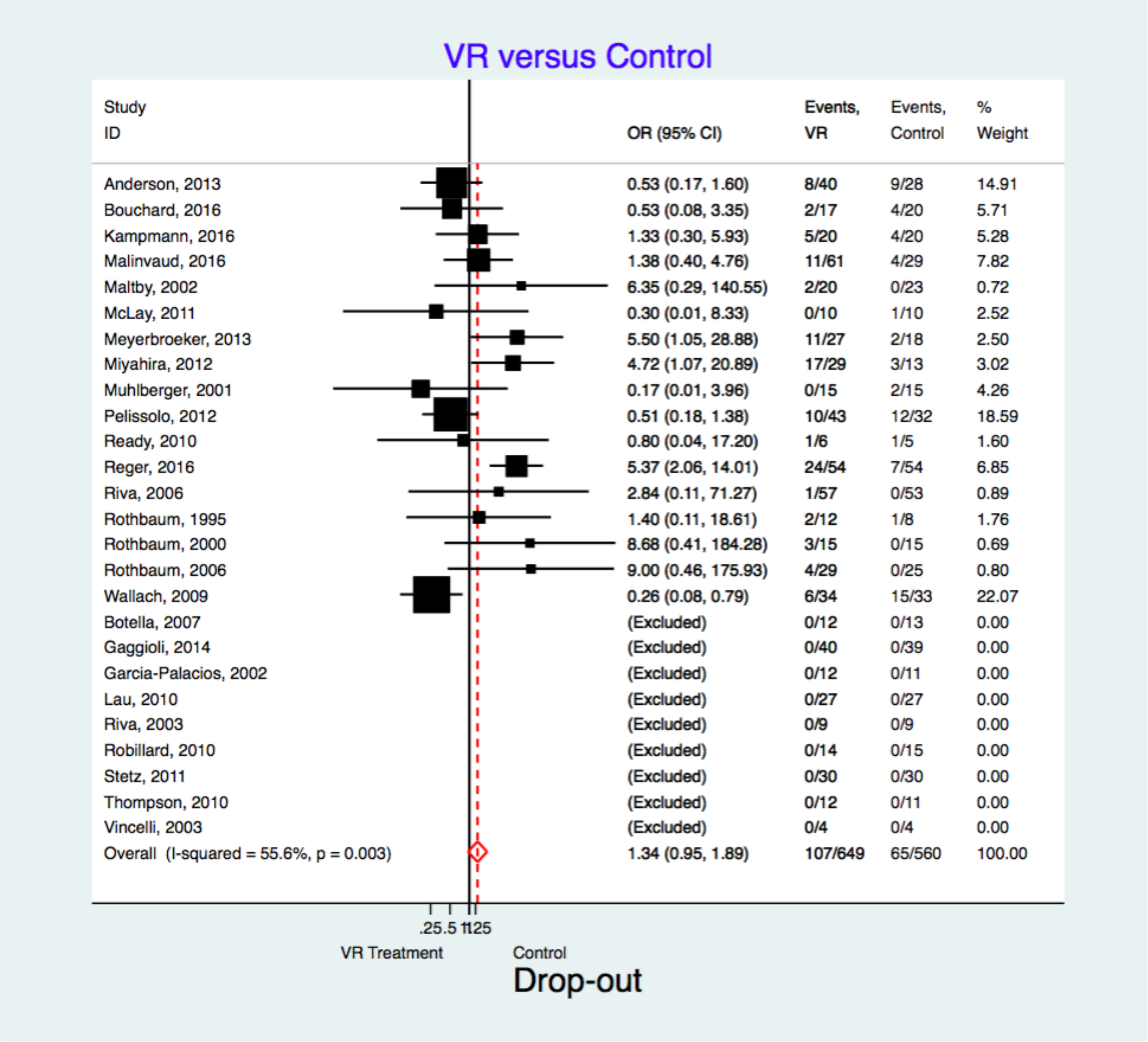

Supplementary Figure S3. Forest plot: Odds ratio post-test for VR-enhanced therapy versus non-VR active psychological treatments for drop-outs

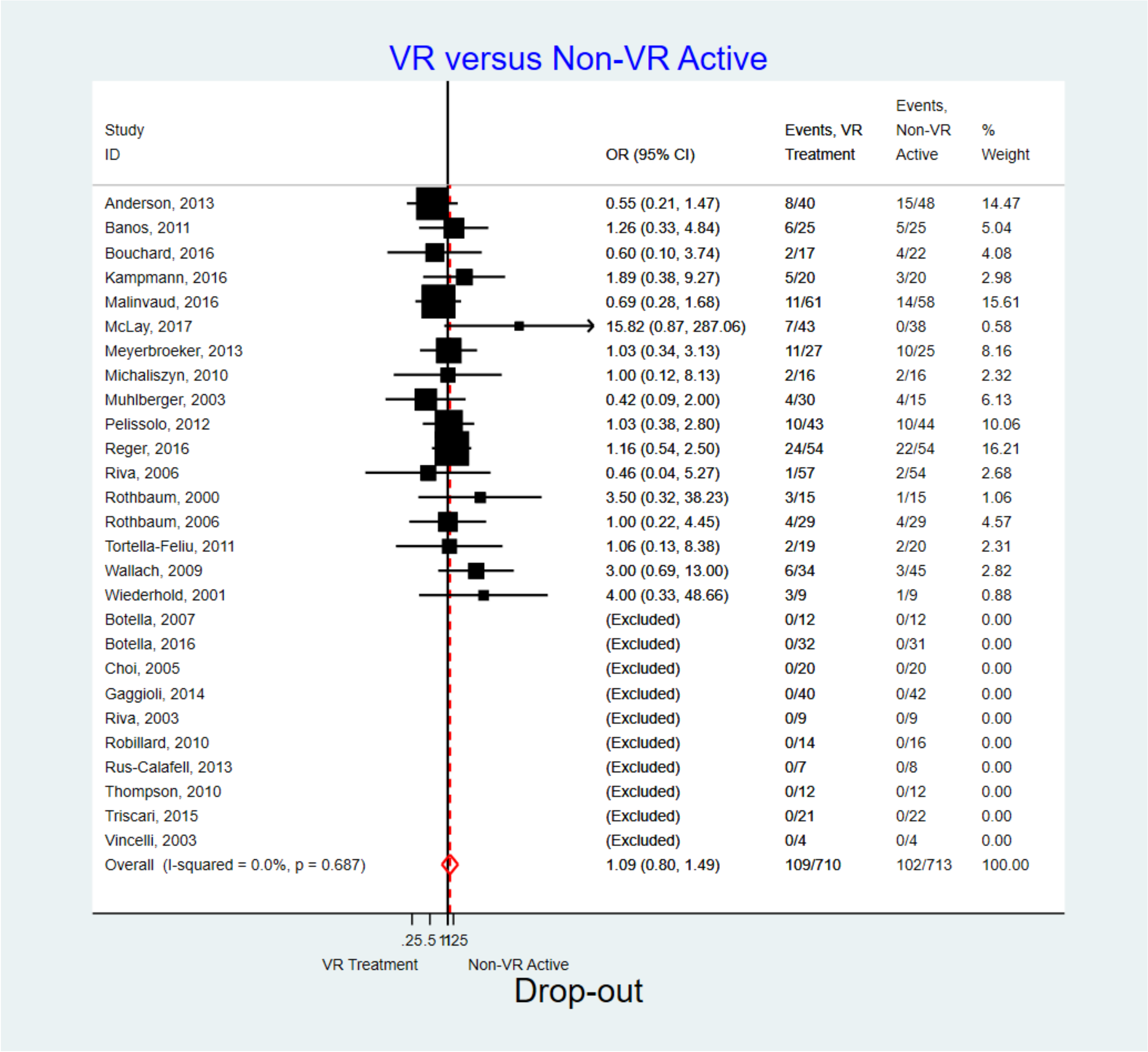

Supplementary Figure S4. Funnel plots for comparison between VR-enhanced therapy and control conditions for depression outcomes: A) Trim and fill adjusted (white circles, observed studies; black circles, imputed studies); B) Contour-enhanced funnel plot; C) Galbraith plot

A

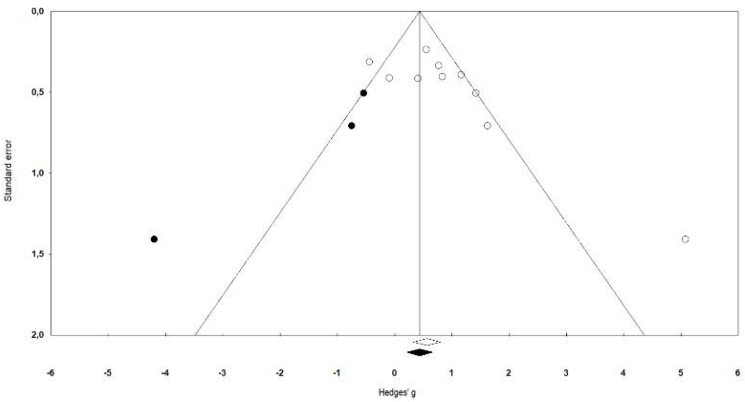

B

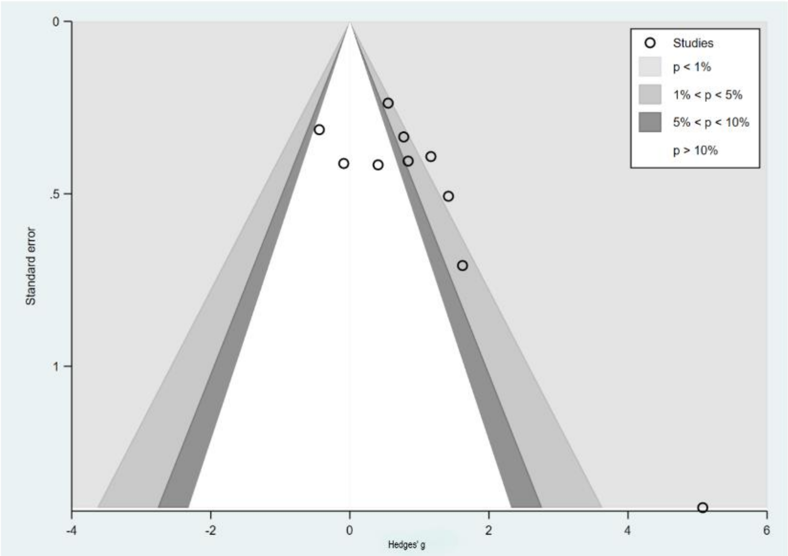

C

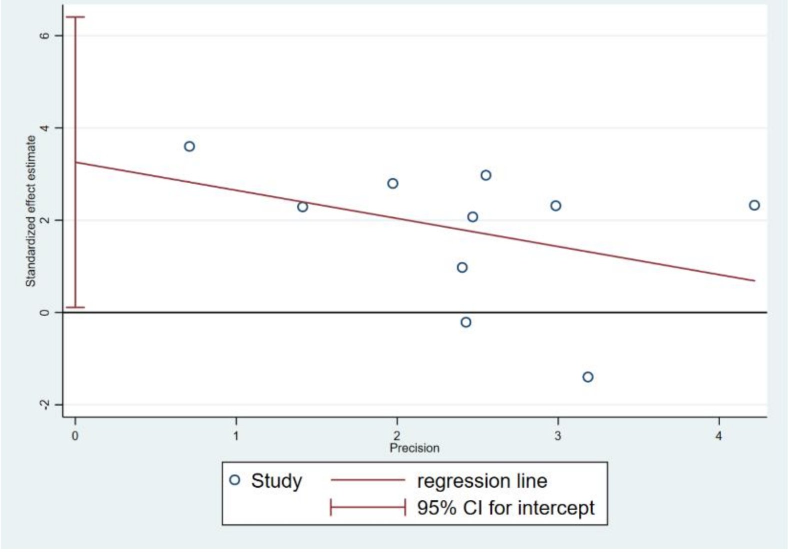

Supplement: Supplementary file 1 — Supplementary information [file 41598_2018_28113_MOESM1_ESM.pdf]
